# Risk Factors of Hypersensitivity to Carboplatin in Patients with Gynecologic Malignancies

**DOI:** 10.3389/fphar.2017.00800

**Published:** 2017-11-06

**Authors:** Yu-Hsiao Tai, Yi-Jou Tai, Heng-Cheng Hsu, Shu-Ping Lee, Yun-Yuan Chen, Ying-Cheng Chiang, Yu-Li Chen, Chi-An Chen, Wen-Fang Cheng

**Affiliations:** ^1^Department of Obstetrics and Gynecology, National Cheng Kung University Hospital, Tainan, Taiwan; ^2^Department of Obstetrics and Gynecology, College of Medicine, National Taiwan University, Taipei, Taiwan; ^3^Department of Obstetrics and Gynecology, National Taiwan University Hospital Hsin-Chu Branch, Hsin-Chu, Taiwan; ^4^Taiwan Blood Services Foundation, Taipei, Taiwan; ^5^Graduate Institute of Oncology, College of Medicine, National Taiwan University, Taipei, Taiwan; ^6^Graduate Institute of Clinical Medicine, College of Medicine, National Taiwan University, Taipei, Taiwan

**Keywords:** ovarian cancer, chemotherapy, carboplatin, hypersensitivity, risk factor

## Abstract

We evaluated the prevalence of and risk factors for hypersensitivity reactions related to carboplatin, which is commonly used to treat gynecological malignancies. All women with pathologically documented ovarian, fallopian tube, or primary peritoneal cancer treated with carboplatin alone or a carboplatin-based combination chemotherapy regimen at a single hospital between January 2006 and December 2013 were retrospectively recruited. We analyzed the incidence, characteristics, risk factors, management, and outcomes of carboplatin-related hypersensitivity reactions among these patients. Among 735 eligible women, 75 (10.2%) experienced a total of 215 carboplatin-related hypersensitivity reaction events. The annual incidence of carboplatin-related hypersensitivity reactions gradually increased from 0.88% in 2006 to 5.42% in 2013. The incidence of carboplatin-related hypersensitivity was higher in patients with advanced stage disease (*P* < 0.001, Kruskal-Wallis test), serous and mixed histological types (*P* = 0.003, Kruskal-Wallis test), malignant ascites (*P* = 0.009, chi-square test), and history of other drug allergy (*P* < 0.001, chi-square test). Compared to women without hypersensitivity reactions, women who experienced hypersensitivity reactions had a significantly greater median cycle number (12 vs. 6, *P* < 0.001, independent sample *t*-test) and dose (6,816 vs. 3,844 mg, *P* < 0.001, independent sample *t*-test). The cumulative incidence of carboplatin-related hypersensitivity reactions dramatically increased with >8 cycles or dose >3,500 mg. Therefore, disease severity, histological type, malignant ascites, past drug allergies, and cumulative carboplatin dose are risk factors for carboplatin-related hypersensitivity reactions. Such reactions could potentially be reduced or prevented by slowing the infusion rate and using a desensitization protocol involving anti-allergy medications.

## Introduction

Ovarian cancer is the leading cause of death from gynecological malignancies (Chiang et al., 2013), with a 5-year survival rate of 46% in the United States (92% when localized, 73% with regional metastases, and 28% with distant metastases) (Siegel et al., 2016). In the majority of ovarian cancer cases, primary management entails surgical staging or cytoreduction, followed by systemic chemotherapy (Ledermann et al., 2013). Platinum-based combination chemotherapy is the recommended treatment for platinum-sensitive recurrent ovarian cancer (Parmar et al., 2003; Pfisterer et al., 2006; Pujade-Lauraine et al., 2010). Parmar et al. (2003) reported that paclitaxel with platinum improves overall survival compared to conventional platinum-based chemotherapy among patients with relapsed platinum-sensitive ovarian cancer. Carboplatin and pegylated liposomal doxorubicin (Pujade-Lauraine et al., 2010) or gemcitabine (Pfisterer et al., 2006) led to superior progression-free survival of patients with platinum-sensitive recurrent ovarian cancer compared to paclitaxel and carboplatin. Therefore, the preferred regimen for platinum-sensitive recurrent ovarian cancer is carboplatin combined with paclitaxel, liposomal doxorubicin, or gemcitabine.

Carboplatin is among the most commonly used chemotherapeutic agents for ovarian, fallopian tube, and primary peritoneal cancer, as it is active against these types of cancers with usually well-tolerated side effects (Ozols et al., 2003; Parmar et al., 2003; Pfisterer et al., 2006; Pujade-Lauraine et al., 2010). However, prolonged carboplatin use is associated with an increased incidence of carboplatin-related hypersensitivity reactions (Markman et al., 1999; Wang et al., 2009). Up to 16% of patients with ovarian cancer who undergo treatment with carboplatin-containing regimens experience carboplatin-related hypersensitivity (Markman et al., 1999; Polyzos et al., 2001; Sliesoraitis and Chikhale, 2005). Though the characteristics of carboplatin-related hypersensitivity reactions are widely reported, limited data are available regarding such reactions in different types of patients.

In Taiwan, carboplatin was first introduced for the treatment of ovarian cancer in 2004. Here, we retrospectively evaluated carboplatin-related hypersensitivity reactions among patients with gynecological cancer (ovarian, fallopian tube, or primary peritoneal cancer) who received carboplatin-containing regimens at a single medical institute in Taiwan. We analyzed the relationships among the incidence, characteristics, risk factors, management, and outcomes of carboplatin-related hypersensitivity reactions in this patient population. We also provide recommendations for the continuation of treatment in ovarian cancer patients.

### Patients and methods

We retrospectively recruited all women with pathologically documented ovarian, fallopian tube, or primary peritoneal cancer treated with single-agent carboplatin or a carboplatin-based combination chemotherapy regimen at National Taiwan University Hospital from January 2006 to December 2013. Patients who previously received platinum compounds were excluded. We retrieved clinical information, including age, menopausal status, cancer stage, surgical findings, chemotherapeutic treatment history, recurrence status, and survivorship, from the clinical and operative notes and discharge summaries stored in a centralized database. The research protocol was reviewed and approved by the institutional review board of the hospital. During the study period, our institution applied carboplatin (including carboplatin with cyclophosphamide or paclitaxel) as the front-line chemotherapy for women with ovarian, fallopian tube, and primary peritoneal cancer. For salvage chemotherapy in the case of recurrent disease, we administered chemotherapeutic regimens including carboplatin with paclitaxel, gemcitabine, or liposomal doxorubicin. Carboplatin-related hypersensitivity reactions were defined as symptoms and signs occurring minutes to hours after carboplatin administration. Such reactions were scored according to the Common Terminology Criteria for Adverse Events version 4.03, with severity assessed as mild (grades 1 and 2) or severe (grades 3 and 4). We also recorded the procedures applied to treat the hypersensitivity reactions and efforts to manage the side effects of carboplatin administration.

Statistical analyses were performed using the Statistical Package of Social Studies (SPSS) version 17.0 (SPSS, Inc., Chicago, IL) and SAS software version 9.4 (SAS Inc, NC, USA) for Windows. Univariate analysis for potential risk factors of carboplatin-related hypersensitivity reactions were assessed by the chi-square or Kruskal-Wallis test for categorical variables. The independent sample *t*-test was used to evaluate how carboplatin cycle and dose correlated with the incidence of hypersensitivity and severe hypersensitivity reactions. Multivariate analysis was conducted using logistic regression to estimate the association between the potential risk factors and the occurrence of hypersensitivity. All the statistical tests were two-tailed, and statistical significant level was defined as *p*-value < 0.05.

## Results

A total of 735 eligible women who underwent treatment for ovarian, fallopian tube, or primary peritoneal cancer were recruited this the study; 75 (10.2%) experienced a total of 215 carboplatin-related hypersensitivity reaction events. The yearly incidence of carboplatin-related hypersensitivity gradually increased from 0.88 in 2006 to 5.42% in 2013 (Table 1), in association with increased use of carboplatin. The total incidence of carboplatin-related hypersensitivity was 3.21% by the cycle of carboplatin.

**Table 1:** The annual incidence of carboplatin-related hypersensitivity reactions in 735 women with ovarian, fallopian tube, or primary peritoneal cancers.

**Year**	**Hypersensitivity (No. of events)**	**Total cycles**	**Incidence (%)**
2006	6	674	0.88
2007	13	860	1.49
2008	23	833	2.69
2009	27	868	3.05
2010	47	914	4.89
2011	42	938	4.29
2012	10	801	1.23
2013	47	820	5.42
2006–2013	215	6,708	3.21

*No, number*.

The characteristics of the 735 patients are presented in Table 2. The median patient age was 56 years (range 16–94 years). Only 2 women are Mongolians and the other 733 women are Asian Taiwanese. A total of 412 (56.1%) patients experienced spontaneous menopause, 295 (40.1%) surgical menopause, and 28 (3.8%) had preserved fertility. Among the 651 women (88.6%) diagnosed with ovarian cancer, 474 (64.5%) had stage III–IV disease. The histological type was serous in 381 patients (51.8%) and clear cell in 148 patients (20.1%). Optimal debulking surgery was performed in 491 patients (66.8%).

**Table 2:** Characteristics of the 735 women receiving carboplatin-based chemotherapy.

**Characteristics**	**Hypersensitivity (*****n*****)**		**Total (*n*)**	**Prevalence (%)**	***p*-value**
	**Positive**	**Negative**			
**AGE (y/o)**					
<45	14	117	131	10.7	0.928
45–65	45	411	456	9.9	
>65	16	132	148	10.8	
**MENOPAUSE**					
Yes Surgical	34	261	295	11.5	0.152
Spontaneous	41	371	412	10.0	
No	0	28	28	0	
**DISEASE ENTITY**					
Ovarian	63	588	651	9.7	0.416
Primary peritoneal	11	65	76	14.5	
Tubal	1	7	8	12.5	
**STAGE**					
I	7	207	214	3.3	<0.001
II	2	45	47	4.3	
III	53	333	386	13.7	
IV	13	75	88	14.8	
**HISTOLOGY**					
Serous	57	324	381	15.0	0.003
Clear cell	6	142	148	4.1	
Endometrioid	6	88	94	6.4	
Mucinous	1	36	37	2.7	
Transitional cell	0	6	6	0	
Carcinosarcomas	1	16	17	5.9	
Mixed type	4	35	39	10.3	
Others	0	13	13	0	
**OPTIMAL SURGERY**					
Yes	43	448	491	8.8	0.066
No	32	212	244	13.1	
**ASCITES (ml)**					
Mild (<500)	40	409	449	8.9	0.232
Moderate (500–1,000)	8	43	51	15.7	
Large (>1,000)	27	208	235	11.5	
**ASCITIC MALIGNANT CELLS**					
Yes	52	353	405	12.8	0.009
No	23	307	330	7.0	
**DRUG OR FOOD ALLERGIC HISTORY**					
Yes	27	117	144	18.8	<0.001
No	48	543	591	8.1	

The rate of hypersensitivity to carboplatin did not differ by patient age, menopausal status, disease entity, receipt of optimal debulking, or the amount of ascites. The rate of hypersensitivity was significantly higher among patients with advanced stage disease (III–IV) compared to patients with early stage disease (I–II) (*P* < 0.001, Kruskal-Wallis test), and among patients with serous or mixed histological type compared to patients with other histological types (*P* = 0.003, Kruskal-Wallis test). We also found higher rates of hypersensitivity among patients with malignant ascites compared to patients without malignant ascites (*P* = 0.009, chi-squared test), and in patients who had experienced allergic reactions to other medications or food (e.g., paclitaxel, penicillin, aspirin) compared to patients who had not experienced previous allergic reactions (*P* < 0.001, chi-squared test). Furthermore, patients with carboplatin hypersensitivity had a higher incidence of previous allergic history to other cytotoxic drugs, including gemcitabine, paclitaxel, and doxorubicin, compared to those without (12/75 vs. 30/660, *P* < 0.001, chi-squared test; Table 7).

**Figure 1:**
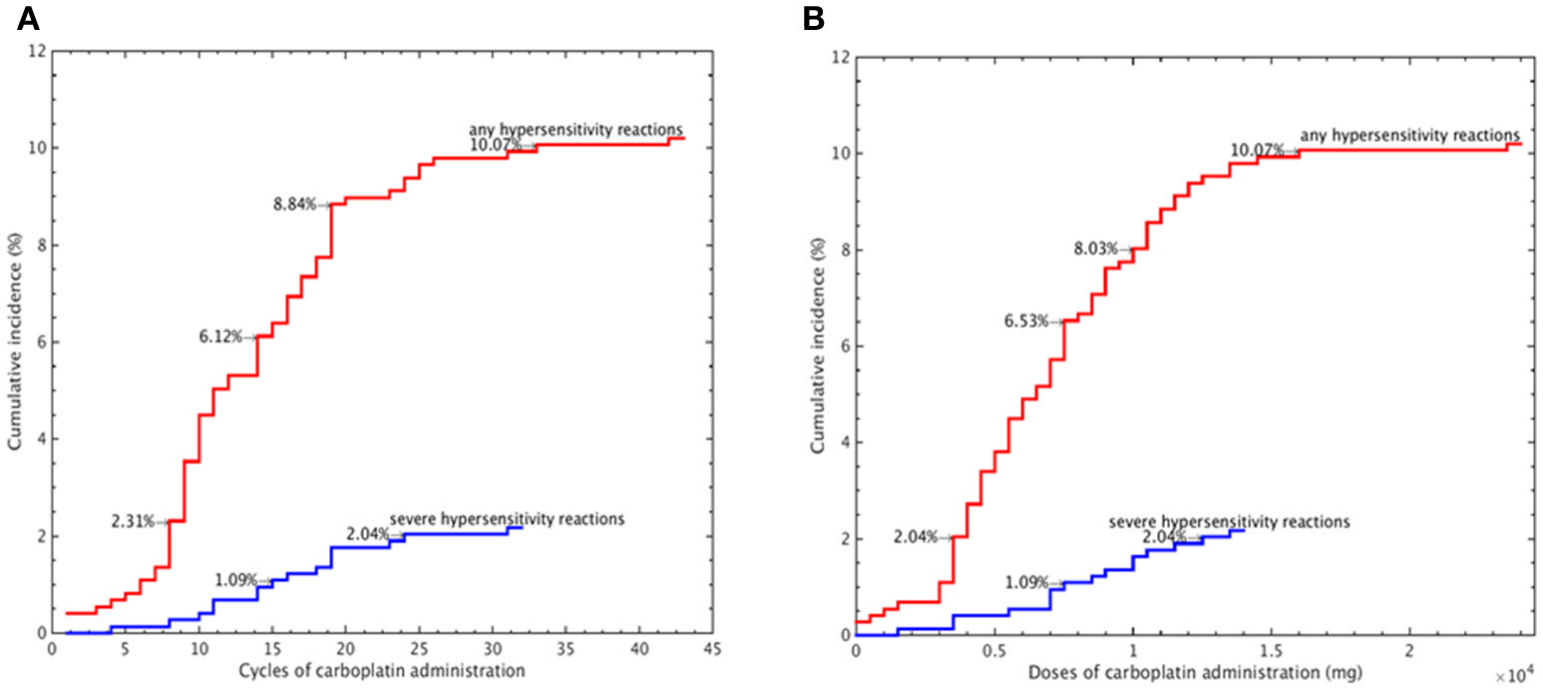
Cumulative incidence of carboplatin-related hypersensitivity reactions according to the carboplatin administration cycle number **(A)** and carboplatin dose **(B)**.

We further evaluated the median carboplatin cycle and dose administered to women with and without carboplatin-related hypersensitivity. Compared to those without hypersensitivity reactions, women who experienced hypersensitivity reactions had a significantly higher median number of cycles (12 vs. 6, *P* < 0.001, independent sample *t*-test) and dose (6,816 vs. 3,844 mg, *P* < 0.001, independent sample *t*-test, Table 3). The cumulative incidence of carboplatin-related hypersensitivity reactions increased with the number of carboplatin cycles (Figure 1A) and increasing dose (Figure 1B), especially at >8 cycles or a dose >3,500 mg. The cumulative incidence of carboplatin-related hypersensitivity was 2% after 8 cycles, 6% after 14 cycles, 8% after 19 cycles, and 10% after 33 cycles, with a plateau beyond this cycle number (Figure 1A). The cumulative incidence of carboplatin-related hypersensitivity was 2% at >3,500 mg, 6% at >7,500 mg, 8% at >10,000 mg, and 10% at >16,000 mg with a plateau beyond this dose (Figure 1B). The cumulative incidence of severe carboplatin-related hypersensitivity was 1% after 15 cycles and 2% after 24 cycles, with a plateau beyond this cycle number, and 1% at >7,500 mg and 2% at >12,500 mg, with a plateau beyond this dose (Figures 1A,B).

**Table 3:** Median cycle and dose of carboplatin administered to women with or without hypersensitivity reactions.

	**Hypersensitivity reactions**		P^*^
	**Yes (*n* = 75)**	**No (*n* = 660)**	
Median cycle (range)	12 (1–42)	6 (1–63)	<0.001
Median dose (mg) (range)	6,816 (328–23,707)	3,844 (150–30,014)	<0.001

N, patient number;

* *By independent sample t-test*.

The symptoms and signs of the 75 women with carboplatin-related hypersensitivity reactions are given in Table 4. All of these reported symptoms and signs occurred within an hour of initiating carboplatin administration. Among these 75 women, 16 (21.3%) experienced severe hypersensitivity reactions (grades 3 and 4). The most commonly exhibited symptom/sign was hypotension (10.7%), followed by anaphylaxis (8.0%) and desaturation (6.7%). A total of 59 women experienced mild signs and symptoms (grades 1 and 2), including skin rash (69.3%), skin itching (64.0%), facial flushing (56.0%), dyspnea (41.3%), chest pain (25.3%), tachycardia (18.7%), paresthesias (16.0%), abdominal cramping (13.3%), and diaphoresis (12.0%). Upon the occurrence of symptoms and signs, carboplatin infusion was immediately stopped. These patients were provided with intravenous fluid infusion and medications, including corticosteroids, antihistamines, and oxygen application. In 74 of the 75 patients, the hypersensitivity reactions subsided within several minutes to several hours after onset, and the patients recovered without any sequelae. Sixty-one of these patients were re-challenged with carboplatin following their initial hypersensitivity reaction. However, 48 (78.7%) of these patients developed repeated hypersensitivity reactions, even after pre-treatment with intravenous antihistamines, H2 blocker, and corticosteroids prior to carboplatin re-administration.

**Table 4:** Symptoms and signs of 75 patients with hypersensitivity reactions to carboplatin.

**Symptoms and signs**	Number of Events^*^ (%)
**MILD REACTIONS**	
Patient number	59
Skin rash	52 (69.3)
Skin itching	48 (64.0)
Facial flushing	42 (56.0)
Dyspnea	31 (41.3)
Chest pain	19 (25.3)
Tachycardia	14 (18.7)
Paresthesias	12 (16.0)
Abdominal cramping	10 (13.3)
Diaphoresis	9 (12.0)
Nausea/vomiting	8 (10.7)
Dizziness	7 (9.3)
Diarrhea	5 (6.7)
Cough	5 (6.7)
Chills	4 (5.3)
Wheezing	2 (2.7)
Fatigue	2 (2.7)
Headache	1 (1.3)
**SEVERE REACTIONS**	
Patient number	16
Hypotension	8 (10.7)
Anaphylaxis	6 (8.0)
Desaturation	5 (6.7)
Seizure	1 (1.3)
Respiratory arrest	1 (1.3)
Died	1 (1.3)

* *Some patients had more than one symptoms and signs*.

**Table 5:** The comparison between other countries and ours about characteristics of the patients receiving carboplatin-based chemotherapy and risk factors of carboplatin-related hypersensitivity reactions.

**Country**	**Taiwan**	**USA (18)**	**Japan (19)**	**Italy (20)**	**Israel (21)**
Number of patients	735	585	113	112	254
Incidence of hypersensitivity reactions	10.2% (75/735)	11.6% (68/585)	8.85% (10/113)	8.0% (9/112)	9.1% (23/254)
Incidence of severe hypersensitivity reactions	2.18% (16/735)	6.67% (39/585)	3.54% (4/113)	2.6% (3/112)	5.91% (15/254)
Death	1	No	No	No	No
Carboplatin hypersensitivity in carboplatin retreatment	61/75	10/68	9/10	n/a	20/23
Median cycle for the first carboplatin-related hypersensitivity reactions	12	n/a	11.5	n/a	9
Median dose for the first carboplatin-related hypersensitivity reactions (mg)	6,816	n/a	8084.5mg	n/a	n/a
Risk factor	Carboplatin cycle or dose Advanced stages (stages III and IV) Serous histologic type malignant ascites Drug or food allergic history	Carboplatin cycle Platinum-free interval of over 12 months Drug allergies	Carboplatin cycle, dose or number of regimens Diagnosis of ovarian cancer	n/a	Carboplatin cycle

*n/a, not available*.

One patient who experienced a carboplatin-related hypersensitivity reaction progressed to respiratory arrest and died. This patient developed hypersensitivity reactions during her 23rd cycle of carboplatin administration at a cumulative dose of approximately 14,000 mg. She experienced skin rash, tachycardia, and dyspnea 13 min after starting the infusion. Tachycardia and dyspnea persisted despite treatment with hydration, antihistamines, corticosteroids, and an oxygen mask. Cardiopulmonary resuscitation and endotracheal intubation were performed, but the patient finally expired due to respiratory failure.

Evaluating whether any factors can predict carboplatin hypersensitivity, we identified a high correlation with the carboplatin cycle and cumulative dose (Pearson correlation coefficient 0.9318). Thus, we chose the cumulative carboplatin dose for the analysis to avoid interference between the highly correlated factors. As shown in Table 6, drug or food history (odd ratios [OR] 2.340, 95% confidence interval [CI] 1.374-3.984, *P* = 0.0018), malignant ascites (OR 1.895, 95% CI 1.108–3.241, *P* = 0.0196), and cumulative carboplatin dose (4,000–4,999 mg: OR 5.507, 95% CI 1.796–16.887, *P* = 0.0028; 5,000–9,999 mg: OR 7.244, 95% CI 2.740–19.149, *P* < 0.0001; ≥10,000 mg: OR 8.461, 95% CI 3.007–23.806, *P* < 0.0001; *P* for trend < 0.0001) significantly positively correlated with carboplatin hypersensitivity.

**Table 6:** Multivariate analyses of clinical parameters for carboplatin-related hypersensitivity reactions.

**Variable**	**Odds ratio (95% confidence interval)**	*P*-value^*^
**AGE, YEARS**		
<65	1	
>65	0.994 (0.538–1.834)	0.983
**DRUG OR FOOD ALLERGY HISTORY**		
No	1	
Yes	2.340 (1.374–3.984)	0.0018
**MALIGNANT ASCITES**		
No	1	
Yes	1.895 (1.108–3.241)	0.0196
**CARBOPLATIN DOSE, mg^#^**		
<3,000	1	
3,000–3,999	2.462 (0.817–7.416)	0.109
4,000–4,999	5.507 (1.796–16.887)	0.0028
5,000–9,999	7.244 (2.740–19.149)	<0.0001
>10,000	8.461 (3.007–23.806)	<0.0001

* *Multivariate analysis*,

# *P for trend < 0.0001*.

**Table 7:** The drug or food allergy in 735 patients receiving carboplatin-based chemotherapy.

**Allergy**	**Carboplatin hypersensitivity**		***P*-value^#^**
	**Yes (*n* = 75)**	**No (*n* = 660)**	
**CONTRAST MEDIUM**			
Yes	5	22	
No	70	638	0.146
**OTHER CYTOTOXIC DRUGS**			
Yes	12	30	
No	63	630	<0.001
**ANTIBIOTICS**			
Yes	5	39	
No	70	621	0.790
**OTHER DRUGS**			
Yes	13	66	
No	62	594	0.052
**FOOD**			
Yes	0	5	
No	75	655	0.450
Total^*^	35	162	

* *One woman may have had an allergy to at least one kind of drug or food*,

# *chi-squared test*.

## Discussion

Our results revealed carboplatin-related hypersensitivity reactions in 1 out of 10 women treated with a carboplatin-containing regimen for ovarian, fallopian tube, or primary peritoneal cancer. This was the largest series of gynecological malignancies investigated for carboplatin-related hypersensitivity reactions. We found a higher incidence of carboplatin-related hypersensitivity among patients with advanced disease (stage III–IV) with serous carcinoma and malignant ascites. These differences were likely related to the fact that patients with advanced disease, serous carcinoma, and ascetic malignant cells required significantly more carboplatin treatment cycles and higher carboplatin doses.

Our findings also suggest that a history of drug or food allergies is a predictive factor for carboplatin-related hypersensitivity. Prior investigations reported that a history of drug allergies correlates with the incidence of carboplatin-related hypersensitivity reactions (Libra et al., 2003; Sliesoraitis and Chikhale, 2005). In our study, 36% of the patients with carboplatin-related hypersensitivity reactions had a history of drug allergies. Patients with prior drug or food allergies should be closely monitored during carboplatin administration.

Table 5 reports the incidences of carboplatin hypersensitivity and patient characteristics reported in various investigations, including the present study. The incidence of carboplatin hypersensitivity ranges from 8 to 11% (Libra et al., 2003; Confino-Cohen et al., 2005; Schwartz et al., 2007; Koshiba et al., 2009). The incidence of severe carboplatin-related hypersensitivity reactions ranges from 2 to 6%, with the lowest incidence occurring in our study. Among cases developing carboplatin-related hypersensitivity reactions, the median number of cycles is 9–12, and the median doses 6,000–8,000 mg. Reported risk factors for carboplatin-related hypersensitivity reactions include carboplatin cycle/dose, platinum-free interval, and history of drug allergies (Libra et al., 2003; Confino-Cohen et al., 2005; Schwartz et al., 2007; Koshiba et al., 2009). Our present findings confirmed these factors and identified other risk factors, including advanced disease, serous histology, and the presence of malignant ascites. These risk factors can help identify patients at greater risk of developing hypersensitivity.

The incidence of carboplatin-related hypersensitivity correlated with cycle number and dose, with the first episode occurring at a median of 12 cycles and 6,816 mg. Similarly, a Japanese study found that carboplatin hypersensitivity occurred at a median of 11.5 cycles and 8,084.5 mg (Koshiba et al., 2009). Markman et al. (Markman, 2007) reported that carboplatin-related hypersensitivity reactions rarely occur before cycle 6 or <3,000 mg of drug delivery, with the first episode most commonly occurring with carboplatin administration in the second-line setting, and carboplatin administration beyond 9 cycles or 6,000 mg increasing the risk of severe hypersensitivity reactions, similar to our present observations. Previous data indicated that prior carboplatin exposure is the primary risk factor for carboplatin-related hypersensitivity reactions among all patients (Navo et al., 2006; Koshiba et al., 2009). Similarly, Kandel et al. (2005) reported that all carboplatin-associated hypersensitivity reactions occur in patients with prior carboplatin exposure, and Tamiya et al. (2011) found that the number of treatment cycles with platinum-containing antineoplastic agents significantly correlates with the incidence of related hypersensitivity reactions. Overall, the evidence supports that the number of carboplatin cycles and dose are the principal risk factors for carboplatin-related hypersensitivity reactions, and that caution is warranted in cases of carboplatin infusion beyond 8 cycles or 3,500 mg.

Severe carboplatin-related hypersensitivity reactions are uncommon. Koshiba et al. (2009) reported a 3.54% rate of severe carboplatin-related hypersensitivity reactions, which is similar to our rate of 2.2%. However, severe carboplatin-related hypersensitivity symptoms can be fatal, as in one of our cases. Thus, the risk of severe carboplatin-related hypersensitivity may influence its usage in gynecological cancers, and it is important to develop protocols to reduce this risk, especially the risk of severe reactions.

Carboplatin-related hypersensitivity reactions sometimes result in premature discontinuation of treatment (Schwartz et al., 2007). One-fifth of these patients never attempt carboplatin reinfusion, even after only mild hypersensitivity reactions (Gadducci et al., 2008). Shah et al. (Bruchim et al., 2014) demonstrated safe re-treatment with carboplatin using a desensitization protocol to reduce repeated hypersensitivity reactions, and Koshiba et al. (2009) reported the successful re-treatment of the majority of patients with previous carboplatin-related hypersensitivity reactions. Altwerger et al. also successfully developed a platinum desensitization protocol for patients with either a positive carboplatin skin test or a history of prior carboplatin hypersensitivity (Altwerger et al., 2017). In the present study, 61 patients with carboplatin-related hypersensitivity were rechallenged with carboplatin. With a lower carboplatin infusion rate and pre-treatment with anti-allergy drugs, all of these patients were able to tolerate re-treatment with carboplatin, although 48 (78.7%) of these 61 patients developed hypersensitivity again despite the desensitization protocol. Several methods have been developed to prevent carboplatin-related hypersensitivity reactions (Libra et al., 2003; Confino-Cohen et al., 2005; Gomez et al., 2009; Greene et al., 2010; Takase et al., 2015; Shah et al., 2016). Genc et al. (2012) demonstrated that carboplatin treatment can be continued successfully in patients whose hypersensitivity reactions are managed in a timely fashion. Abe et al. (2010) described the use of cisplatin as an alternative to carboplatin. However, other studies have reported severe hypersensitivity reactions to cisplatin in patients with prior carboplatin hypersensitivity reactions (Zweizig et al., 1994; Dizon et al., 2002).

Carboplatin induces DNA double strand breaks, which are repaired by BRCA recombination repair enzymes. BRCA1/2 mutation has been reported to correlate with carboplatin hypersensitivity (Altwerger et al., 2017). Furthermore, Moon et al. reported that the majority (93%) of patients with a BRCA1/2 mutation develop carboplatin hypersensitivity, and that BRCA1/2 mutation is an independent risk factor for the development of carboplatin hypersensitivity. (Moon et al., 2013) BRCA1/2 mutation patients had early-onset carboplatin hypersensitivity at a lower cumulative exposure. The BRCA status of our patients was not available for further analysis in this retrospective study. However, basophil activation has been observed in patients with a history of severe carboplatin hypersensitivity reaction (Iwamoto et al., 2014). Whether, BRCA 1/2 and HRD-related genes modulate Th2 gene expression and increase specific IgE to carboplatin is still unknown. Recently, more and more patients with ovarian cancer have been receiving genetic counseling and testing, including BRCA1/2 and the other heterologous recombinant deficient (HRD) genes, because of the development of PAPP inhibitors. It would be worthwhile to evaluate the correlation between other HRD genes and carboplatin hypersensitivity.

A history of drug or food allergy, the presence of malignant ascites, and the cumulative carboplatin dose are three independent predictive factors of carboplatin hypersensitivity in this survey. A cumulative carboplatin dose >4,000 mg significantly positively correlated with carboplatin hypersensitivity. Close monitoring is warranted for patients with any one of these three risk factors who are receiving carboplatin. Reducing the carboplatin infusion rate and employing desensitization protocols with anti-allergy medications are especially important for patients at high risk of carboplatin hypersensitivity to detect the hypersensitivity early and avoid severe hypersensitivity without compromising the efficacy of the antineoplastic regimen. It would be worthwhile to design a perspective trial that could randomize patients with carboplatin hypersensitivity to receive an alternative regimen without carboplatin. The results of the trial could answer whether carboplatin hypersensitivity patients have similar chemo-responses in non-platinum regimens without compromising the efficacy of the antineoplastic regimens of these patients.

In conclusion, in our present population of women with gynecological malignancies treated with platinum-based chemotherapy, 1 in 10 developed carboplatin-related hypersensitivity reactions. Risk factors for these reactions included advanced disease, serous histological type, malignant ascites, and history of drug allergies. Close monitoring is warranted for patients with a history of drug or food allergy, malignant ascites, or cumulative carboplatin dose >4,000 mg. Strategies for preventing carboplatin-related hypersensitivity include reducing the carboplatin infusion rate and employing desensitization protocols with anti-allergy medications. In addition, the development of a new generation of platinum cytotoxic drugs to avoid hypersensitivity reactions is warranted.

## Ethics statement

This study was approved by the Research Ethics Committee at the National Taiwan University Hospital (201706023RINC).

## Author contributions

Study conception and design: Y-HT and W-FC. Acquisition of data: Y-HT, S-PL, Y-CC, Y-LC. Analysis and interpretation of data: Y-HT, Y-JT, H-CH, Y-YC, and W-FC. Drafting of manuscript: Y-HT and W-FC. Critical revision: C-AC.

### Conflict of interest statement

The authors declare that the research was conducted in the absence of any commercial or financial relationships that could be construed as a potential conflict of interest.
